# Refining Lung Cancer Screening Criteria in the Era of Value-Based Medicine

**DOI:** 10.1371/journal.pmed.1002226

**Published:** 2017-02-07

**Authors:** Steven D. Shapiro

**Affiliations:** Department of Medicine, University of Pittsburgh School of Medicine and UPMC, Pittsburgh, Pennsylvania, United States of America

## Abstract

In a Perspective on the Research Article by ten Haaf and colleagues, Steven Shapiro discusses the challenges of identifying screening strategies that maximize the number of cancers diagnosed, while minimizing the harms of overdiagnosis and maintaining cost-effectiveness.

Lung cancer remains the leading cause of cancer mortality worldwide, and while mortality is gradually decreasing in high-income countries for most cancers, lung cancer mortality is not decreasing and is actually increasing in women [1]. Moreover, effective treatment for advanced stages of lung cancer remains elusive, suggesting a great need for early detection, if indeed efforts to prevent onset of smoking and rapid cessation fail. In 2011, the National Lung Screening Trial (NLST) demonstrated that screening for lung cancer with three annual chest CT scans in smokers (or those who quit within 15 years) of at least 30 pack years, between 55–74 years old, reduced mortality by 20% compared to a single chest radiograph [2]. As important as this finding was, in an era of excessive and rising health care costs, it is necessary to carefully assess cost-effectiveness and refine screening criteria to maximize value. In this issue [3], ten Haaf and colleagues applied microsimulation modeling to 576 different scenarios to determine the population-based cost effectiveness of lung cancer CT screening, using the Canadian health care system threshold ($50,000 Canadian dollars per life-year gained) as a benchmark. The optimal screening scenario thus identified included smokers (or those who quit <10 years prior) with a smoking history of at least 40 pack years to be screened annually from ages 55–75. They estimate that such a screening strategy would reduce mortality 9.05% compared to no screening at an incremental cost-effectiveness ratio of $41,136 Canadian dollars (US$33,825, in 2015) per life-year gained. While they estimate this strategy would not catch as many lung cancers as using the criteria from the NSLT trial would, they predict the optimal strategy would be more cost-effective and would reduce expected false positive screens and lung cancer overdiagnosis compared to the NSLT criteria. The future goal will be to increase efficacy without additional cost.

The desire to eliminate cancer is, in part, at odds with the move to value-based medicine. Value—defined as outcomes relative to cost—cannot be ignored, as health care occupies a large and increasing proportion of most nations’ gross domestic product, particularly the United States. This battle has been playing out for several tumor types. For example, the United States Preventive Services Task Force (USPSTF) recently suggested that screening mammography for breast cancer begin for the general population of women at age 50 [4], but the American Cancer Society [5] and other medical specialty organizations continue to press for earlier initiation of screening. Debate also rages as to the age at which screening should stop, given the limited life expectancy of elderly patients and that some cancers may show a different, less aggressive, biology in late life.

The hard fact remains that when cost-effectiveness enters into the decision of whether to screen, some cancers will be missed that otherwise could have been caught early and cured. To maximize the benefit of screening strategies, studies such as the one in this issue are needed to carefully model the contribution of relevant factors such as burden of cigarette smoking, time from cessation, age of the patient, and testing frequency in order to catch as many tumors as possible within a feasible price and harm limit. Cost-effectiveness threshold like the one used in this manuscript—modeled to be effective if disease-related costs per life-year gained were less than $50,000 Canadian dollars—is reasonable, albeit somewhat arbitrary, and may be best determined by a nation’s societal values and capability. Nevertheless, beyond a certain monetary amount, the cost to society will, in practical implementation, outweigh the individual benefit. Beyond societal necessity, overtesting can also harm the individual. Both radiation and, in the case of false positive imaging results, invasive procedures can cause unnecessary morbidity and mortality. With respect to CT screening for lung cancer, the traditional cancer risk-averse approach to potentially malignant lesions (“when in doubt take it out”) will result in more surgical procedures, thereby increasing noncancer risk, as mass screening will greatly increase the number of tests performed and incidental nodules detected. In order to further refine risk–benefit estimates, the harms of screening therefore require further evaluation.

How do you maximize the ability to detect as many early lung cancers as possible while limiting unnecessary testing and associated cost and harm? One possible approach lies in precision medicine to personalize care [6]. The ability to capture “panomic” big data (genetic, genomic, metabolomics, etc.) in addition to social and demographic data combined with machine learning algorithms will allow focus on screening those most susceptible to lung cancer. Moreover, advances in imaging—such as molecular imaging—and ability to capture circulating tumor cells may enhance capacity for early diagnosis beyond the CT scan.

With respect to lung cancer screening, the hope is that big data can pinpoint a smaller number of patients at significant risk for developing malignancy that can be targeted for aggressive screening. It is important not to lose sight of the fact that if smoking were eliminated, then lung cancer would be an orphan disease. Improvement of diet and exercise would also reduce the burden of many chronic diseases, but these behaviors are notoriously difficult to modify and will require personalized care in their own right, in addition to public health initiatives and better promotion within health insurance programs. Fine-tuning of screening strategies and advances to imaging techniques will improve early diagnosis and the chances of effectively treating lung cancer. At the same time, continued focus on healthier lifestyle choices will bring upstream benefits to individual well-being and national economic health.
